# General symptom reporting in female fibromyalgia patients and referents: a population-based case-referent study

**DOI:** 10.1186/1471-2458-9-402

**Published:** 2009-10-31

**Authors:** Karin Björkegren, Mari-Ann Wallander, Saga Johansson, Kurt Svärdsudd

**Affiliations:** 1 Department of Public Health and Caring Sciences, Family Medicine and Clinical Epidemiology Section, Uppsala University Hospital, Uppsala, Sweden; 2 AstraZeneca R&D Mölndal, Department of Epidemiology, Mölndal, Sweden; 3 Department of Medicine, Sahlgrenska University Hospital/Östra, Gothenburg, Sweden

## Abstract

**Background:**

Fibromyalgia is characterized by widespread musculoskeletal pain and palpation tenderness. In addition to these classic symptoms, fibromyalgia patients tend to report a number of other complaints. What these other complaints are and how often they are reported as compared with related referents from the general population is not very well known. We therefore hypothesized that subjects with fibromyalgia report more of a wide range of symptoms as compared with referents of the same sex and age from the general population.

**Methods:**

138 women with diagnosed fibromyalgia in primary health care and 401 referents from the general population matched to the cases by sex, age and residential area responded to a postal questionnaire where information on marital status, education, occupational status, income level, immigrant status, smoking habits physical activity, height and weight history and the prevalence of 42 defined symptoms was sought.

**Results:**

The cases had lower educational and income levels, were more often unemployed, on sick leave or on disability pension and were more often first generation immigrants than the referents. They were also heavier, shorter and more often had a history of excessive food intake and excessive weight loss. When these differences were taken into account, cases reported not only significantly more presumed fibromyalgia symptoms but also significantly more of general symptoms than the referents. The distribution of symptoms was similar in subjects with fibromyalgia and referents, indicating a generally higher symptom reporting level among the former.

**Conclusion:**

Subjects with fibromyalgia had a high prevalence of reported general symptoms than referents. Some of these differences may be a consequence of the disorder while others may reflect etiological processes.

## Background

Fibromyalgia is a syndrome characterized by widespread musculoskeletal pain and increased palpation tenderness. Fibromyalgia prevalence has been estimated to two to four percent in the general population, [1-3] with strong female predominance [1,4]. The syndrome is chronic, with little or no healing tendency. There are many hypotheses regarding etiology.

According to the American College of Rheumatology (ACR) 1990 criteria, the fibromyalgia diagnosis is based on tender points in a minimum of 11 of 18 specific sites and widespread pain, i.e., axial pain plus pain above and below the waist and in the right and left side of the body and for at least 3 months [5,6]. In addition, numerous other symptoms, such as fatigue, sleep disturbances, morning stiffness, irritable bowel syndrome (IBS) [5,7-9], headache, psychological distress [8,9] and subjectively impaired cognitive function [8,10], are commonly reported. Laboratory findings are usually normal [6]. Moreover, fibromyalgia patients have difficulties in performing activities of daily living [11], affecting their quality of life negatively [12], they have limited work capacity and are often on disability pension [11]. This circumstance and their frequent use of health care resources have negative financial consequences in relation to public health costs [1,9,11,12].

Most of the symptoms mentioned above have been linked to the fibromyalgia condition, although they are not strictly part of the current classification criteria. However, it might be that fibromyalgia patients have more widespread symptoms than generally believed, which might be evidence that fibromyalgia has its origin in the nervous system rather than in the musculoskeletal. We therefore decided to test the hypothesis that women with fibromyalgia, in addition to traditional fibromyalgia symptoms, report more of a wide range of general symptoms than women in the corresponding age segment of the general population in a case-referent study large enough to allow for adjustments for potential confounders or other symptom prevalence affecting variables.

## Methods

### Study population

The study was performed in Uppsala county, central Sweden. All patients in the county who fulfil the 1990 ACR criteria [5] for fibromyalgia diagnosis are offered referral to a fibromyalgia patient educational team, run by one of the authors (KB), with including medical and physiotherapy competence at a primary health care center in the city of Uppsala. All fibromyalgia patients in the county who fulfill the criteria are entered into a fibromyalgia patient register and are offered a rehabilitation programme. The vast majority of all diagnosed fibromyalgia patients in the county are included in the program.

From this register, the 150 most recently entered female patients were sampled. For each case five referents, matched to the cases by age, sex and residential area (postal code), were sampled from the national population register, which is required by law to be kept up-to-date. The population register includes all residents of Sweden, whether citizens or not, and persons are identified by name, address, and a unique personal identification number that includes information on date of birth and sex. The matched case-referent groups were numbered consecutively (referred to as match number below). A questionnaire, presented as a general health survey, was mailed to all the 900 cases and referents, of which 138 (92.0%) cases and 401 (53.5%) referents responded after one reminder when necessary. Overall there were responses from cases and at least one matched referent in 90.0% of case-referent matched groups.

### Data collection

From the postal questionnaire, information was obtained regarding marital status, number of children and number of children still in the household, working status, educational background, household income, immigrant status, smoking habits, physical activity at work and during leisure time, height, weight and weight history, presence of presumed fibromyalgia and other symptoms, and menstrual status.

Marital status was classified as never married, married or cohabiting, divorced or widowed. Occupational status was classified as working full time outside the home, or being a student, working part time, unemployed, on sick leave for more than six months, retired because of disability, old age pensioner, or other status. Highest attained educational level was classified as compulsory (secondary) school only, vocational training, secondary school, or college or university education. Annual household income from work before taxes was given at the one of five possible levels shown in Table 1 (1 SEK approximately equalling 0.14 US$ or 0.10 €). If the respondent was a first generation immigrant, the country of origin was requested, and for second generation immigrants the country of origin for each of the parents.

**Table 1:** Study population

	**Cases**		**Referents**				
					
	**Mean or %**	**SD**	**Mean or %**	**SD**	**p**	**OR**	**95%CI**
N	138		401				
Age, years	49.5	8.79	48.8	8.25	n.s.	0.97	0.93-0.997
Marital status, %					n.s.		
Single	7.4		9.3				
Married or cohabiting	71.1		73.4				
Divorced	18.5		15.0				
Widowed	3.0		2.3				
No. of children	2.4	1.16	2.2	1.28	n.s.		
Migrant status, %							
1st generation immigrant	20.3		9.7		< 0.001	2.39	1.40-4.06
2nd generation immigrant	2.9		4.0		n.s.		
Education, %					< 0.001	0.80	0.65-0.98
Secondary school only	22.0		8.8				
Vocational training	17.7		16.5				
High school	31.6		23.3				
College or university	28.7		51.4				
Occupational status, %					< 0.001	3.58	2.63-4.86
Working full time	10.3		52.1				
Working part time	15.4		26.4				
Unemployed, on sick-leave or retired	74.3		19.4				
Other	-		2.1				
Income level (SEK/year), %					< 0.001	0.90	0.75-1.08
< 100.000	16.1		5.2				
100,000-149,000	19.7		8.3				
150,000-199,000	12.2		11.6				
200,000-299,000	19.9		28.7				
> 300.000	32.1		46.2				
Pain duration, years	16.0	11.8	-	-			

Characteristics of the study population.

Smoking habits were classified as never smoked, ex-smoker, currently smoking less than 15 grams per day, smoking 15-24 grams per day, or smoking 25 or more grams per day, one cigarette equalling 1 gram, a cigarillo 2 grams and a cigar 5 grams. Wet snuff-taking habits were classified accordingly, one portion equalling 1 gram. Leisure time physical activity was classified into four levels ranging from sedentary lifestyle to vigorous physical activity [13]. Physical activity at work was classified as sedentary, physically mobile work, or physically heavy work [13].

Information on height was sought to the nearest centimetre and weight to the nearest kilogram. Body mass index (BMI) was calculated as weight in kilograms divided by height in metres squared. In addition, information was sought on recalled weight at age 20, weight five years ago, highest weight ever attained, the respondent's ideal weight, whether the respondent had experienced periods of perceived underweight, or periods of excessive food intake, and the weight at that time. Furthermore, the question "How is your weight distributed?" was asked with response alternatives "on the belly/upper part of the body", "on the hips/thighs" and "equally in both locations" to obtain information on the subjects' body mass distribution.

**Figure 1:**
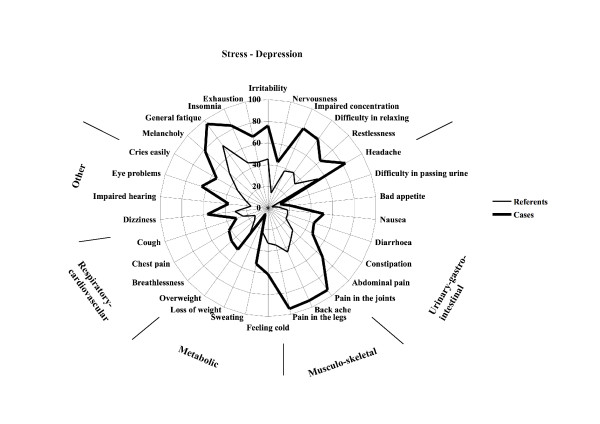
**General symptom prevalence**. Polar diagram showing the prevalence of 30 general symptoms experienced during the past three months among fibromyalgia patients and age-sex-residential area matched referents from the general population. The symptoms are arranged according to symptom groups.

Two sets of symptom reporting questionnaires were used. General symptoms were measured with the Complaint Score sub-scale of the Gothenburg Quality of Life Instrument [14]. The instrument contains a list of 30 general symptoms, listed in figure 1. The respondent was asked to indicate which of these symptoms she had experienced during the past three months with possible responses "yes" (= 1) or "no" (= 0). The symptoms form six symptom groups. The scores are summed to an overall Complaint Score, possible range 0-30, and may also optionally be summed across the symptom groups. The instrument is not intended to measure the prevalence of specific diseases but rather the tendency to report symptoms.

Based on presumed fibromyalgia symptoms listed in previous publications [1,5,11,15], a symptom list including the twelve most common and a few atypical symptoms was compiled. The respondent was requested to indicate what symptoms she experienced during the past 3 months. In addition, for those reporting pain the pain history duration, measured in years, was sought. Information on menstrual status was based on a question about whether the menstruation had ceased, and if so, what year the last menstrual period occurred.

There were no differences between respondents and non-respondents regarding age or place of residence in the total study population or within the case group and reference group, respectively.

The study was performed in accordance with the Helsinki Declaration. All respondents gave their informed consent to participation in the study. The Research Ethics Committee at the Medical faculty, Uppsala University, approved the study (Ups 01-088).

### Statistical analysis

The analyses were performed with the SAS software [16]. Summary statistics such as means and dispersion measures were computed according to conventional parametric methods. Simple differences between the two groups in continuous variables were tested with Student's t-test and differences in proportions with the chi-square test.

The analyses of differences in symptom reporting were performed with logistic regression analysis, producing odds ratios and their 95% confidence intervals (95%CI), with the symptom entered as dependent variable (outcome) and the group variable (case or referent) as the independent variable. To adjust for the influence on outcome of the significant differences in characteristics between the groups, these (immigrant status, education, occupational status, income, and BMI) were entered as covariates in the analyses, with backward elimination of non-significant covariates. All analyses were conditional, i.e., cases were compared only with their own referents, to avoid bias owing to the variable number of responding referents per case. This was achieved by using the match number as an additional covariate in the analyses. Only two-tailed tests were used. P-values of less than 5% were regarded as statistically significant.

## Results

### Characteristics of the study population

There were no differences between the groups regarding age, marital status, or number of children, Table 1. The cases were first generation immigrants significantly more often than the referents, and had, on average, less education. A lower proportion of cases than referents had a job, and cases had lower income than referents. There were no differences between the groups in proportion of postmenopausal women or mean age at menopause.

Lifestyle variables, weight, and weight history are shown in Table 2. The cases were similar to the referents in tobacco use, physical activity and body mass index (BMI) at age twenty. Present height, weight, and BMI differed between the groups, with the cases being significantly shorter and heavier than the referents. There was no difference in dominating body mass distribution (waist or hips/thighs) between the groups. Significantly more cases than referents reported underweight or excessive food intake ever.

**Table 2:** Medical history

	**Cases**		**Referents**				
					
	**Mean or %**	**SD**	**Mean or %**	**SD**	**p <**	**OR**	**95%CI**
N	138		401				
Tobacco use, %					n.s.	1.08	0.89-1.31
Never smoked	34.6		45.0				
Ex-smoker	43.4		31.0				
Current smoker							
1-14 g/day	14.7		14.8				
15-24 g/day	4.4		7.4				
≥25 g/day	2.9		1.8				
Taking snuff	4.4		3.1				
Low physical activity, %							
During leisure time	86.6		80.3		n.s.	0.99	0.72-1.35
During work	50.0		43.8		0.001.	0.24	0.18-0.33
Height, m	1.65	0.06	1.67	0.06	0.05	0.96	0.93-0.99
Weight, kg							
At age 20	55.9	7.3	57.7	7.8	0.05	0.97	0.94-0.99
Five years ago	69.7	12.5	57.7	7.8	0.05	1.02	1.00-1.03
At present	72.8	13.9	68.1	11.8	0.0005	1.03	1.01-1.05
Body mass index, BMI							
At age 20	20.6	2.5	20.8	2.5	n.s.	0.97	0.89-1.05
Five years ago	25.7	4.6	24.1	4.3	0.0005	1.08	1.04-1.13
Maximum	28.9	5.3	26.4	5.2	0.0001	1.08	1.05-1.12
At present	26.8	4.9	24.6	4.1	0.0001	1.11	1.07-1.16
Body mass distribution, %					n.s.		
Waist	24.0		19.2			1.22	0.75-1.98
Hips/thighs	12.8		19.4			0.64	0.36-1.15
Both sites equal	63.2		61.4			1.00	-
Ever had underweight, %	35.6		24.5		0.05	1.70	1.12-2.59
Excessive food intake, %	17.9		8.0		0.005	2.53	1.42-4.49

Lifestyle variables, weight and weight history, fibromyalgia duration and menstrual history.

### Symptom reporting

The prevalence rates of presumed fibromyalgia symptoms during the past three months are displayed in Table 3. As expected, the cases had a significantly higher prevalence of all symptoms, also when the influence on symptom reporting of the covariates immigrant status, education, occupational status, income, and BMI was taken into account (p < 0.0001). The odds ratios ranged from a low 4.10 for muscular fatigue to a high 31.33 for daily dull aching.

**Table 3:** Fibromyalgia symptoms

	**Cases**	**Referents**	**OR**	**95%CI**	**p**
Constantly exhausted, %	89.0	34.7	10.67	5.78-19.69	< 0.0001
Whole body fatigue, %	91.9	36.9	16.31	7.84-33.93	< 0.0001
Only muscular fatigue, %	46.3	15.6	4.10	2.62-6.43	< 0.0001
Tender points, %	95.6	40.2	26.63	11.37-62.38	< 0.0001
Sense of stiffness, %	95.6	42.0	25.23	10.75-59.20	< 0.0001
Sense of bloating, %	72.1	22.1	6.30	3.81-10.43	< 0.0001
Numbness, pins and needles, %	77.2	26.4	5.48	3.27-9.19	< 0.0001
Wakes up not thoroughly rested, %	86.0	45.2	6.95	4.10-11.78	< 0.0001
Deteriorated short-term memory, %	73.5	28.9	4.66	2.86-7.59	< 0.0001
Daily pain, dull aching, %	94.9	24.4	31.33	13.82-71.03	< 0.0001
Pain at rest, %	94.9	27.9	26.26	11.61-59.40	< 0.0001
Awakened because of pain, %	83.8	19.4	13.15	7.47-23.13	< 0.0001

Prevalence of fibromyalgia symptoms among cases and referents. OR = odds ratios adjusted for the influence of age, body mass index, education, occupational and immigrant status, 95%CI = 95% confidence intervals.

The mean number of reported symptoms in the Complaint Score instrument was 17.7 (SD 5.89) among the cases and 8.9 (SD 5.41) among the referents when the influence of the covariates was taken into account (p < 0.0001). As shown in Table 4 and Figure 1, the Complaint Score difference was not confined to any special symptom or symptom group. Generally, cases appeared to report symptoms more often than referents, the differences between the groups being significant for 25 of the 30 symptoms.

**Table 4:** General symptoms

	**Cases**	**Referents**	**OR**	**95%CI**	**p**
Cries easily, %	54.7	32.4	1.92	1.20-3.06	< 0.01
Melancholy, %	78.1	47.9	3.04	1.85-5.00	< 0.0001
General fatigue, %	95.6	71.1	9.44	4.03-22.12	< 0.0001
Insomnia, %	83.2	45.4	5.96	3.66-9.73	< 0.0001
Exhaustion, %	67.2	43.1	2.79	1.85-4.21	< 0.0001
Irritability, %	75.9	45.1	4.25	2.71-6.69	< 0.0001
Nervousness, %	43.1	14.2	3.16	1.93-5.18	< 0.0001
Impaired concentration, %	80.3	37.4	7.11	4.43-11.40	< 0.0001
Difficulty in relaxing, %	78.1	40.4	5.51	3.49-8.71	< 0.0001
Restlessness, %	65.0	33.2	3.92	2.59-5.93	< 0.0001
Headache, %	82.5	53.4	4.40	2.69-7.18	< 0.0001
Difficulty in passing urine, %	11.7	3.7	1.88	0.85-4.16	0.12
Bad appetite, %	21.2	9.5	1.56	0.85-2.84	0.15
Nausea, %	51.8	18.2	4.92	3.19-7.59	< 0.0001
Diarrhoea, %	44.5	19.0	3.43	2.26-5.22	< 0.0001
Constipation, %	47.4	19.0	3.86	2.54-5.87	< 0.0001
Abdominal pain, %	67.9	30.7	5.03	3.29-7.67	< 0.0001
Pain in the joints, %	93.4	35.7	17.60	8.33-37.19	< 0.0001
Back ache, %	93.4	44.4	16.70	8.22-33.93	< 0.0001
Pain in the legs, %	94.9	34.9	21.96	9.71-49.68	< 0.0001
Feeling cold, %	61.0	32.4	2.36	1.46-3.82	0.0005
Sweating, %	52.6	23.2	3.28	2.14-5.03	< 0.0001
Loss of weight, %	6.6	5.5	0.87	0.36-2.07	0.75
Overweight, %	47.4	27.7	1.51	0.92-2.49	0.10
Breathlessness, %	45.3	16.5	2.44	1.47-4.05	0.0005
Chest pain, %	41.6	13.7	3.85	2.43-6.10	< 0.0001
Cough, %	30.7	24.2	1.19	0.76-1.86	0.46
Dizziness, %	56.2	30.7	2.90	1.95-4.32	< 0.0001
Impaired hearing, %	36.5	16.0	3.00	1.93-4.68	< 0.0001
Eye problems, %	64.2	21.7	6.48	4.25-9.89	< 0.0001

Prevalence of general symptoms among cases and referents. OR = odds ratios adjusted for the influence of age, body mass index, education, occupational and immigrant status, 95%CI = 95% confidence intervals.

## Discussion

The cases thus had more symptoms than the referents, not only regarding fibromyalgia symptoms as expected, but also regarding symptoms in general as measured with the Complaint Score instrument, also when differences between cases and referents in immigrant status, education, occupational status, income, and BMI were taken into account.

The study was performed as a postal questionnaire study in patients fulfilling the 1990 ACR criteria and in a matched referent group sampled from the general population. The response rate among the cases was 92% and among the references a moderate 54%. However, in the case-referent matched groups 90% had a responding case and 92% at least one responding referent. Since the analyses were conditional, i.e., the cases were systematically compared with their own referents, and all referents for a case are inter-changeable by definition, the response rate within the case-referent groups was satisfactory. It may be argued that the response among referents was selective. If so, the respondents were most probably healthier than non-respondents. On the other hand, the responding referent group had the same proportion of fibromyalgia patients as the corresponding age segment of the general population, i.e. approximately 12 subjects. We also know from another of our studies [17], that the Complaint Score level among the referents in the present study was similar to what is found in the corresponding segment of the general population. All things considered, the responding referents are most probably representative of the general population.

The questionnaires used were all validated except for the fibromyalgia symptom list, which was used as an instrument for the first time in this study. However, the list contains only well-known fibromyalgia symptoms. The recall time frames were generally short, 14 days to one year, with the exception of weight history. However, it has been shown that recall of height [18] and weight [19] at age 20 corresponds well with the corresponding medical record data. We therefore have no reason to believe that there would be selection or measurement bias to such an extent that the conclusions would be affected.

A number of descriptive studies on fibromyalgia patients have been presented. White et al. [11] investigated 100 fibromyalgia patients diagnosed according to the ACR criteria, 135 referents, age and sex matched to the fibromyalgia patients, and 76 patients with widespread pain of other origin. Mean age was 48 years, and 86% were women. Compared with referents, fibromyalgia patients tended to have lower income and educational levels, while there were no differences in marital status or reproductive history. As in our study, fibromyalgia patients reported more overall symptoms, more severe pain and fatigue, and worse overall health.

Henriksson and Liedberg [20] studied 176 female fibromyalgia patients, whose mean age and educational level were about the same as in our study. Fifty percent were still working, as compared with 26.6% among our cases. Wolfe et al. [21] studied work disability in 1,604 individuals with fibromyalgia, of whom 89% were women, mean age 48 years, and 42% were employed.

The present study appears to be the first controlled study based on random samples of cases and referents in which the higher prevalence is contrasted with the reporting in the corresponding age and sex segment of the general population. The distribution of reported general symptoms was the same in cases and referents, but the cases had a significantly higher prevalence of almost all symptoms. The higher general symptom prevalence among the cases may be due either to a higher true prevalence or to individuals with fibromyalgia having no more general symptoms than the general population but being more attentive to bodily symptoms. It is debatable whether fibromyalgia is merely an extension of the usual aches and pains of the general population, or whether the neuro-endocrine stress and pain systems are triggered, making patients more susceptible to experiencing and reporting symptoms.

A true increase in symptom prevalence may be specific to fibromyalgia or unspecific and attributable to the presence of a chronic disease. In a recent study there was no significant difference between coronary heart disease patients and matched controls in Complaint Score reporting, indicating that chronic disease per se does not increase general symptom reporting [17]. In previous fibromyalgia studies, some controlled but none based on random samples, fibromyalgia patients had more general symptoms than rheumatic arthritis patients [22-25] and than patients with osteoarthritis and other pain syndromes [11,23,24], making the possibility that the increased symptom prevalence was caused by chronic disease per se less likely. In any case, symptoms reported by subjects with fibromyalgia remain quite stable over the years, as has been shown in a longitudinal study [26].

Cases were immigrants to a significantly higher extent than referents. This may be explained in terms of the difficulties immigrants experience related to entering the labour market, because of language problems, etc. This circumstance might perhaps be involved in the aetiology of the condition.

There were also significantly more individuals among the cases who had ever suffered from underweight or excessive food intake. This was an unexpected finding. Individuals with fibromyalgia often have gastro-intestinal disturbances. There is a known co-morbidity between fibromyalgia and irritable bowel syndrome [27] and between fibromyalgia and rheumatoid arthritis [24,25]. It is not known whether there is a connection between the gastro-intestinal disturbances seen in patients with fibromyalgia and eating disorders, or whether eating disorders, per se might induce fibromyalgia, and the cause of the link fibromyalgia-rheumatoid arthritis as well appears to be unknown.

The diagnosis of fibromyalgia is usually based on the ACR criteria, which are quite specific and may be too narrow. The ACR criteria include specified tender points and widespread pain. However, individuals with fibromyalgia often have more general symptoms in addition. In a number of other conditions the symptom criteria are divided into A and B symptoms, for instance in chronic fatigue syndrome, lymphomas, and others. Using the same reasoning in fibromyalgia might then mean that the ACR criteria would constitute A symptoms and other reported symptoms, such as those in the present study, would constitute B symptoms.

## Conclusion

In this case-referent study, the first controlled study of general symptom reporting in fibromyalgia patients based on random samples of cases and referents, we found significantly more frequent reporting of general symptoms among the patients than among referents. The cause may be either that fibromyalgia patients are more conscious of symptoms that others ignore or that fibromyalgia patients have a higher true prevalence of symptoms than referents, and thereby more symptoms than has generally been recognised.

## Competing interests

SJ is and MAW was at the time of the data collection employees of AstraZeneca R&D Mölndal. However, AstraZeneca had no drug on the market relating to the content of the manuscript. The study was partly funded by AstraZeneca R&D Mölndal.

## Authors' contributions

All authors participated in the data collection process and design of the data analysis. KB and KS performed the analyses and wrote the manuscript draft. All authors participated in the discussions of the results and in the revisions of the manuscript.

## Pre-publication history

The pre-publication history for this paper can be accessed here:


